# Using WeChat to guide preparation before transthoracic echocardiography reduces anxiety and improves satisfaction of parents of infants with congenital heart disease

**DOI:** 10.1186/s13019-023-02225-1

**Published:** 2023-05-09

**Authors:** Shan Guo, Wen-Hao Lin, Shi-Hao Lin, Qi-Liang Zhang, Hua Cao, Qiang Chen

**Affiliations:** 1 Department of Ultrasound, Fujian Children’s Hospital (Fujian Branch of Shanghai Children’s Medical Center), College of Clinical Medicine for Obstetrics & Gynecology and Pediatrics, Fujian Medical University, Fuzhou, China; 2 Department of Cardiac Surgery, Fujian Children’s Hospital (Fujian Branch of Shanghai Children’s Medical Center), College of Clinical Medicine for Obstetrics & Gynecology and Pediatrics, Fujian Medical University, Fuzhou, China

**Keywords:** WeChat, TTE, CHD, Anxiety, Satisfaction

## Abstract

**Objective:**

To explore the effect of using WeChat to guide preparation before transthoracic echocardiography (TTE) on reducing anxiety and improving the satisfaction of parents of infants with congenital heart disease (CHD).

**Methods:**

This study was a retrospective study conducted in a children’s hospital. The clinical data of 44 patients and the anxiety and satisfaction data of their parents who received WeChat guidance were collected between December 2021 and January 2022 (the WeChat group). The corresponding data of 47 patients and their parents who received educational brochure guidance were collected between September 2021 and November 2021 (the routine group). Guidance was used to help the parents prepare for TTE performed by medical professionals. The State-Trait Anxiety Inventory scale and the Patient Satisfaction Questionnaire-18 (PSQ-18) were used. The data of the two groups were compared and analyzed.

**Results:**

The comparison of parental anxiety between the two groups showed that the scores of state anxiety and trait anxiety in the WeChat group were significantly lower than those in the routine group (p < 0.05). The comparison of the results of the PSQ-18 showed that the scores for general satisfaction, interpersonal manner, communication, time spent with the physician, and accessibility and convenience in the WeChat group were significantly higher than those in the routine group (p < 0.05).

**Conclusion:**

Using WeChat to guide preparation before TTE for infants with CHD can effectively reduce the anxiety of their parents and improve their parents’ satisfaction with medical treatment.

## Background

Transthoracic echocardiography (TTE) is the most important examination to evaluate the condition of children with congenital heart disease (CHD) [1]. With the development of echocardiography technology, most CHD treatment decisions have been made according to echocardiography results [2, 3]. Comprehensive and accurate echocardiography results require the close cooperation of patients and doctors [4]. However, infants and young children are often unable to cooperate in completing this examination. Sedatives are routinely used in many centers for infant examination [5, 6]. Due to inadequate preparation, sedation and TTE examination often fail, and parents usually have a fearful attitude toward the use of sedatives [7, 8]. Therefore, many families of patients feel very anxious about undergoing TTE [9]. Because of the lack of knowledge of echocardiography and sedation, they are often unable to prepare well for the examination, which results in repeated sedation, repeated examination or even examination failure. It is very important to provide effective preparation guidance before TTE for parents of patients to improve the success rate of examination, reduce the anxiety of parents and improve their satisfaction with medical treatment. We conducted this retrospective study to explore the effect of using WeChat to guide preparation before TTE on reducing anxiety and improving the satisfaction of parents of infants with CHD.

## Methods

The present study was approved by the ethics committee of our hospital and adhered to the tenets of the Declaration of Helsinki. Additionally, all parents of the patients signed the consent form before participating in the study.

This study was a retrospective study conducted in a children’s hospital. Based on the differences in the State-Trait Anxiety Inventory (STAI) scores of parents from the pre-experiment and assuming that the alpha value was set at 0.05 with a power of 0.80, the required number of participants was calculated to be 40 in each group. Assuming a 10% drop-out rate, the total sample size was set as 88 (44 per group). The following inclusion criteria were used: (1) infants with CHD who underwent TTE as an outpatient service; and (2) family members who could easily use the internet and WeChat. The following exclusion criteria were used: (1) infants with CHD who underwent emergency TTE; (2) infants with other congenital diseases; and (3) family members of the patients refused to participate in the study.

Through the electronic medical record system, we retrospectively collected the clinical data of 44 patients and the anxiety and satisfaction data of their parents who received WeChat preparation guidance before TTE between December 2021 and January 2022. The clinical data of 47 patients and the anxiety and satisfaction data of their parents who received preparation guidance before TTE via the routine educational brochure between September 2021 and November 2021 were retrospectively collected as a routine group. Parents were routinely asked to complete the STAI scale and the Patient Satisfaction Questionnaire-18 (PSQ-18) after the patient underwent TTE, and these data were collected and stored. All patients were given 0.5 ml/kg 10% chloral hydrate before TTE.

### WeChat guidance methods

We began to use WeChat to guide parents of patients to prepare for TTE in December 2021. After making an appointment for TTE, the medical staff instructed the patient’s parents to join the WeChat group and taught them to use WeChat correctly and skillfully. The content of WeChat guidance mainly included two parts: an education module and answering questions. The education module included TTE-related knowledge and matters needing attention, sedation-related knowledge and matters needing attention and so on. Parents could read and learn at their convenience. In the answering questions module, a medical staff member was on duty every day and was online at a particular time, explaining the consultation to the parents and repeatedly reminding the patients of the TTE examination process, fasting and sleep deprivation before sedation.

Before December 2021, we guided parents of patients to prepare for TTE via an educational brochure. Therefore, the parents in the routine group received a brochure after booking the time of TTE that included the same content as the education module in the WeChat group. They were told to read carefully after going home and prepare for the examination in advance.

### Research tools

The STAI, which was compiled by Spielberger, contains 40 items and measures transient and enduring levels of anxiety. Studies in different populations showed that indicators of internal consistency supported the reliability of the factors and subscales, and the interfactor correlations reflected positively on the concurrent validity of the different STAI factor and subscale measures. This scale contained 20 items for both STAI state anxiety and STAI trait anxiety. The first 20 items were STAI state anxiety, assessing anxiety of the individual during an immediate or recent specific anxious time. Half of the items described negative emotions, and the other half described positive emotions. The next 20 items were STAI trait anxiety, assessing anxiety during a stable time. Among them, 11 items described negative emotions, and 9 items described positive emotions. All items were scored by 4 grades: 1 = little, 2 = a few degrees, 3 = middle degree, and 4 = almost always. The higher the score, the more obvious the anxiety was [10, 11].

The PSQ-18 questionnaire was used to assess parents’ satisfaction. The eighteen questions of the questionnaire consisted of several subscales, including general satisfaction, technical quality, interpersonal manner, communication, financial aspects, time spent with the physician, and accessibility and convenience. The questions were scored using a five-point Likert scale from 1 to 5 [12].

### Statistical analysis

SPSS19.0 software was used for statistical analysis. The quantitative data are expressed as the mean ± standard deviation, and the continuous data were normally distributed. A t test was used for statistical analysis, and a chi-square test was used to compare the qualitative data between the two groups. P < 0.05 was considered statistically significant.

## Results

The demographic data of all patients and their parents are shown in Table 1, and there was no significant difference in the demographic data between the two groups. Comparison of parental anxiety data between the two groups showed that the scores for state anxiety and trait anxiety in the WeChat group were significantly lower than those in the routine group (p < 0.05). Comparison of the PSQ-18 results showed that the scores for general satisfaction, interpersonal manner, communication, time spent with the physician, and accessibility and convenience in the WeChat group were significantly higher than those in the routine group (p < 0.05). There was no significant difference in technical quality and finance scores between the two groups (P > 0.05) (Table 2).

**Table 1:** Demographic characteristics of patients and their parents in two groups

	The WeChat group (n = 44)	The routine group (n = 47)	P
Age (month)	6.1 ± 2.7	6.3 ± 2.5	0.738
Weight (kg)	6.2 ± 1.6	6.4±.1.5	0.516
Disease			
Ventricular septal defect	14	17	0.908
Patent ductus arteriosus	11	14	
Pulmonary stenosis	6	4	
Tetralogy of fallot	2	1	
Coarctation of the aorta	2	3	
Atrial septal defect	9	8	
Age of parents (year)	27.9 ± 4.3	28.6 ± 4.5	0.440
Parents’ education level			
Under high school	15	18	0.929
High school	15	13	
Junior college	8	9	
Bachelor degree or higher	6	7	
Living condition			
Rural area	32	35	0.851
City	12	12	

**Table 2:** Comparison of parental anxiety and satisfaction between the two groups

	The WeChat group (n = 44)	The routine group (n = 47)	P
The preoperative state anxiety score	33.0 ± 6.6	37.6± 9.7	0.011
The preoperative trait anxiety score	33.1± 4.5	36.7 ± 8.0	0..010
General satisfaction	3.9 ± 0.8	3.4± 0.8	0.002
Technical quality	3.8± 0.9	3.7± 0.8	0.248
Interpersonal manner	3.8 ± 0.9	3.4± 0.9	0,034
Communication	4.9 ± 0.8	3.1± 0.9	0.001
Financial aspects	3.9± 0.9	3.6 ± 0.8	0.184
Time spent with doctor	3.9± 0.8	3.4 ± 0.7	0.002
Accessibility and convenience	4.2± 0.6	3.0± 0.8	0.001

## Discussion

CHD is the most common and serious congenital structural malformation in infancy and childhood [13, 14]. TTE is the most commonly used method to evaluate the patient’s condition and develop a treatment strategy for CHD. High-quality and accurate cardiac ultrasound results are not only related to the ability of doctors and medical equipment but also closely related to the cooperation of patients during examination. However, infants with CHD who are usually incapacitated are unable to cooperate with the completion of TTE examination and often need to be sedated [5, 6]. Therefore, to successfully complete TTE, it is necessary to prepare for the examination and sedation, including fasting and sleep deprivation. Otherwise, it may lead to failure of the examination and repeated sedation. In particular, advanced medical care is mainly concentrated in large cities in our country; only a few provincial hospitals can perform cardiac surgery, and the level of cardiac ultrasound examination in these hospitals is also trustworthy [15]. As a result, most infants with CHD need to go to these hospitals for TTE. The patients usually need to make an appointment in advance, so their parents hope to successfully complete the examination on the same day of the appointment. Otherwise, having to repeat the examination and making another appointment will take too much time and energy. Most of the patients in this study (73.8%) lived in rural areas, and most of their parents (67%) were educated below the junior college level. They had little medical knowledge, and they could not master this knowledge well by reading simple brochures alone. They were worried that inadequate preparation would lead to failure in the examination and were very anxious. In addition, the parents of infants with CHD had more stress, anxiety and depression than the general population. These parents were more likely to have bad psychological emotions even before the cardiac examination. Therefore, it is very important to explore a more effective guidance method for these parents before TTE.

In recent years, with the development of mobile information technology, smartphones have been widely used all over the world, and telemedicine is increasingly widely used [16]. Many studies have shown that remote health education is more effective than traditional information transmission methods in shortening time consumption, reducing economic costs, improving treatment compliance, reducing complications, increasing follow-up rates and improving patients’ conditions [17–19]. Studies by Liu and Kang have shown that the application of mobile multimedia in intestinal preparation education for colonoscopy could improve the quality of intestinal preparation and reduce the cancellation rate of planned colonoscopy [20, 21]. Liu and other studies have shown that the use of mobile multimedia in preoperative preparation for daytime surgery could effectively increase parents’ knowledge about perioperative preparation and promote their children’s preparation for daytime surgery, thus reducing the chance of canceling surgery [16]. A prospective randomized study conducted by Wang et al. also showed that the use of mobile media for guidance before colonoscopy could improve the quality of intestinal preparation and improve the detection rate of tumors [22].

As the most widely used social platform in China, WeChat is an all-in-one communication application with text, voice, video calls and photo sharing, with more than 1 billion users in the country [23]. Our previous studies have shown that the use of WeChat as a health education tool can effectively reduce the preoperative and postoperative care burden and anxiety of parents and improve their quality of life [24–26]. In this study, WeChat was used to guide parents to prepare for TTE examinations. The families of the WeChat group could learn from WeChat’s education module anytime and anywhere according to their own needs. If they had any questions, they could consult the medical staff through the WeChat platform anytime and anywhere, and they could obtain timely and effective answers and the support of professional knowledge, which could effectively improve parents’ understanding of TTE knowledge and matters needing attention and fully prepare them for the examination. At the same time, we also encouraged the family members of the WeChat group to communicate with each other and share their successful cases and experiences so that parents could feel the power of collective struggle and enhance their confidence and hope. Through WeChat, we could also understand the psychological state of parents in a timely manner; listen to patients and provide care, guidance and support; alleviate negative psychology; and eliminate worries. The results of this study also showed that parents’ anxiety in the WeChat group was significantly lower by providing advance guidance through the WeChat platform.

The satisfaction of patients and their families is also an important index to evaluate medical services [27]. Through the WeChat platform, we could conveniently and effectively provide remote high-quality medical services of the hospital to family members so that they could easily obtain medical support without leaving home and alleviate the fear and pressure brought by the disease to families. Without increasing medical costs, it could effectively improve the satisfaction of parents with medical services. The results of this study also showed that, through WeChat-provided advance guidance, the parents in the WeChat group had significantly higher satisfaction with health care.

There were still some limitations in this paper. First, this was a retrospective study, and the statistical power was not as good as that of a prospective study. Therefore, a prospective controlled study is necessary in the future to further validate our conclusions. Second, this was a single-center study, and there might be a certain deviation in the selection of cases. Therefore, it is necessary to conduct multicenter, multiarea collaborative research in the future. Third, parents without the internet or smartphones were unable to participate in this study because of the demand for equipment. Fourth, the research indicators adopted in this study were all subjective indicators without objective indicators. We do not have statistics on the total dose of sedatives used, examination length, quality and completeness difference, or examination failure rate, which would affect the results to some extent. Different studies might come to different conclusions. Therefore, a larger sample size is needed to further verify the accuracy of the conclusion.

## Conclusion

Using WeChat to guide preparation before TTE for infants with CHD can effectively reduce the anxiety of their parents and improve their parents’ satisfaction with medical treatment.

## Data Availability

The datasets used and analyzed during the current study are available from the corresponding author on reasonable request.
